# Hydrogen permeation through steel during cathodic polarization of lubricating oils in a modified Devanathan–Stachurski cell

**DOI:** 10.1038/s41598-022-21941-7

**Published:** 2022-11-04

**Authors:** Tz. Boiadjieva-Scherzer, L. Mirkova, G. Fafilek, J. Reinbold, H. Kronberger, H. Stache, G. Bodesheim, M. Monev

**Affiliations:** 1 Centre of Electrochemical Surface Technology GmbH (CEST GmbH), Viktor-Kaplan-Straße 2, 2700 Wiener Neustadt, Austria; 2 Institute of Physical Chemistry, Bulgarian Academy of Sciences, Acad G. Bonchev Str., Bl.11, 1113 Sofia, Bulgaria; 3 TU-Vienna, Institute for Chemical Technology and Analytics, Getreidemarkt 9/164ec, 1060 Vienna, Austria; 4 Klüber Lubrication München GmbH & Co. KG, Geisenhausenerstrasse 7, 81379 Munich, Germany

**Keywords:** Chemistry, Materials science

## Abstract

In lubricated tribo-contacts, hydrogen ingress in steel is possible due to chemical reactions of lubricant components like base oils or additives, and/or contamination upon service particularly water, and/or corrosion processes, and/or electrostatic fields or current flow. Absorbed by the metal, atomic hydrogen may cause serious deleterious effects on the physical–chemical and mechanical properties, reducing the material’s ability to withstand the design loads. The present research work is focused on analyzing the influence of electric field on lubricating oils in contact with steel surface. In order to evaluate the possibility of atomic hydrogen generation and permeation into the steel under cathodic polarization of lubricating oils the electrochemical permeation technique developed by Devanathan and Stachurski is used. The input cell of a Devanathan–Stachurski set up is appropriately modified by realizing a very close distance between the working electrode (steel membrane) and a Pt counter electrode with the oil between. This significantly increases the sensibility of the set up and allows the application of larger voltage and higher temperature to enable hydrogen generation from lubricating oils. The complex effects of cathodic polarization, temperature, additives and presence of water in model lubricating oils on atomic hydrogen permeation into steel is discussed.

## Introduction

Hydrogen permeation into metals may cause very serious deleterious effect, well known as “hydrogen embrittlement”, on their physical–chemical, structural and mechanical properties. This phenomenon has stimulated studies of various aspects aimed to its understanding, prediction and prevention.

Hydrogen embrittlement is caused by penetration of H atoms into metals. The hydrogenation of the metals leads to decrease in the ductility of the metal, deformation under applied stress, cracking and blisters. Hydrogen can be incorporated in the metal during various stages of manufacturing processes as well as during its use. The problem is most important in the case of iron and steel constructions^1^.

The phenomenon “hydrogen embrittlement” is widely investigated mainly in aqueous media^2–5^. The kinetics of hydrogen permeation into metals depends on many factors such as the nature of the metals and their surface state, especially the presence of oxides, applied potential or current density in the case of electrochemical charging, pH, chemical composition of the solution and additives which may inhibit or promote the hydrogen permeation^1,3,6–14^.

Various experimental methods for evaluation of the hydrogen coverage, respectively hydrogen permeation into metals are proposed in the literature. Among them, the electrochemical technique, derived by Devanathan–Stachurski is widely used^15,16^. It provides rapid, reliable and precise information about hydrogen absorption and diffusivity. Suitable modifications of Devanathan–Stachurski cell were also developed to investigate hydrogen permeation through metals at different hydrogen entry conditions.

The phenomenon “hydrogen embrittlement” of steel constructions used in non-aqueous media is also investigated.

It is reported that the steel bearings used in various machines, in particular in power trains, often suffer from a short service life due to flaking caused by the structural changes. Various factors are proposed as a cause of the problem of the brittle flaking accompanying peculiar structural changes in steel during the rolling contact. Among them are vibration^17^, bending stress^18^, chemical composition of the lubricant^19,20^ and current flow through the bearings^21,22^.

To answer what is the source of hydrogen in bearings and which factors prevent or promote hydrogen ingress, several hypotheses are proposed. Some of them support the basic idea that this phenomenon occurs when hydrogen formed after the decomposition of the lubricating oils by a tribochemical reaction diffuses into the steel bearings^19,20^. According to other authors^23^, in normal bearing operation, hydrogen is considered to come mainly from the reaction of water (as water contaminants from the air humidity) at the bearing surface along with the decomposition of the lubricant. It is found in this study that the fatigue life of the steel bearings is inversely proportional to the hydrogen content of the steel. Possible source of hydrogen could be also the diffusion of hydrogen absorbed during the bearing manufacturing process.

In the modern industry of lubricating oils, substances that are a complex mixture of organic compounds (base oil and additives) are commonly used. Some of the additives provide antioxidant, anti-corrosion, anti-wear, anti-friction and water-resistant properties^24^.

A mechanism of formation of atomic hydrogen from decomposition of lubricant molecules, additives or contaminants via tribochemical reactions under the tribological contact during surface friction is proposed as a result of investigations with a space lubricant under vacuum conditions^25^. After adsorption of the lubricating oil on the steel surface, the cleavage of C–C bonds follows, which results in the release of hydrogen and hydrocarbon gases.

It is suggested that the oils, composed of hydrocarbons, decompose under conditions of high temperature and repeated stress caused by rubbing contact and generate hydrogen^23,25^. Thus, hydrogen ingress into the steel occurs due to changes within the oil composition upon service. As a result, flaking of the steel bearings is observed. Thermal decomposition of chemisorbed water and surface contaminants participating in the tribochemical processes occurring at the interface are the other possible sources of hydrogen evolution^25^. The effect of the high temperature on the stability of the oil substances, formation of hydrogen and following micro structural changes of the steel induced by the hydrogen embrittlement is established and well described in Ref.^26^. Controlling the diffusion of hydrogen can effectively prevent the flaking and provide long bearing life.

To evaluate the hydrogen uptake from a lubricated tribo-contact into bulk steel, in-situ hydrogen detection methods are developed by suitable modification of the Devanathan–Stachurski set up^27–29^. By replacing the charging side of the conventional cell with a “lubricated rubbing” device, and by recording H-permeation transients on the detection side, the effect of commonly used additives and water contamination of the lubricating oils on hydrogen permeation into steel is investigated^28^.

Early bearing failures, well known as white etching cracks (WEC), are observed when the rolling contact is subjected to so-called additional load such as electrical currents flowing through the bearings, in addition to the pure rolling load^21,22,30,31^. WEC are networks of cracks below the surface of the raceway in the white etching areas. This phenomenon is one of the undesirable side effects during the operation of bearings. It leads to a drastic reduction in the bearing service life. The influence of small currents passing through the rolling bearings on formation of WEC is investigated in detail in Ref.^32^. On the basis of the results, the failure hypothesis “cathodic WEC fatigue” is suggested as follows: a direct current flow through a rolling bearing causing a small voltage drop can lead to very strong electrical fields (> 10 kV/mm) due to the extremely thin lubricating gaps. Relatively low current densities (10^–6^–10^–4^ A/mm^2^) could be sufficient for this. Depending on the lubricant composition and a sufficient voltage, electrochemical reactions take place and possibly also the formation of solvated hydrogen cations (protons). According to the bearing tests, a voltage drop across the bearing of approximately 1 V seems to be sufficient. At critical hydrogen concentrations of less than 1 ppm WEC formation begins.

No reports in the literature on the effect of external polarization of lubricating oils on the hydrogen permeation in in situ measurements were found.

It could be assumed that depending on the electrical currents passing through the rolling bearings and the lubricant composition, electrochemical reactions take place. As a result, formation and permeation of hydrogen into the steel bearings can be expected. The present work was motivated to study the influence of electric current on lubricating oils in contact with steel surface. The objectives of this work are:

- Development of a modification of the Devanathan–Stachurski cell for detection and analysis of the hydrogen permeation into steel membrane in contact with lubricating oils subjected to cathodic polarization.
- Investigation of the influence of the cathodic polarization on the hydrogen permeation behaviour of various oil compositions (including water contamination) at room temperature and upon heating.

Model oil compositions, including commonly used in lubricating oils additives as Calcium Sulfonate (CaSulf) and/or Zinc-dialkyldithiophosphate (ZDDP) were chosen for the method validation. Based on literature, it is considered that both CaSul and ZDDP are critical in driving WEC formations, as one specific factor is the effect of the additives’ chemistry on the generation and diffusion of hydrogen^20,30–32^.

## Experimental

Preliminary investigations of the hydrogen permeation into steel membrane in the presence of lubricating oils using the conventional Devanathan–Stachurski set up showed the necessity of a suitable modification of the input (charging) cell in order to facilitate the passing of the charging current or voltage through the oil and to increase the hydrogen permeation sensibility. The reason is the low conductivity of the lubricating oils.

The input side of the conventional Devanathan–Stachurski cell was redesigned to achieve very close distance between the working electrode (steel membrane) and Pt counter electrode of the input cell^33^. In this modified version, the output (detection) cell is the same as in the classical version.

The working electrode and Pt counter electrodes are placed between two polymer rings, which after assembling form a large ring (modified input cell). To establish a close distance between the steel membrane and the Pt counter electrode, a thin spacer of filter paper (blue line, with a thickness of 130 µm) impregnated with the oil is used. The experiments were performed under room temperature or upon heating. At the experiments upon heating, the input cell was connected with a heating device through the Pt counter electrode.

**Figure 1:**
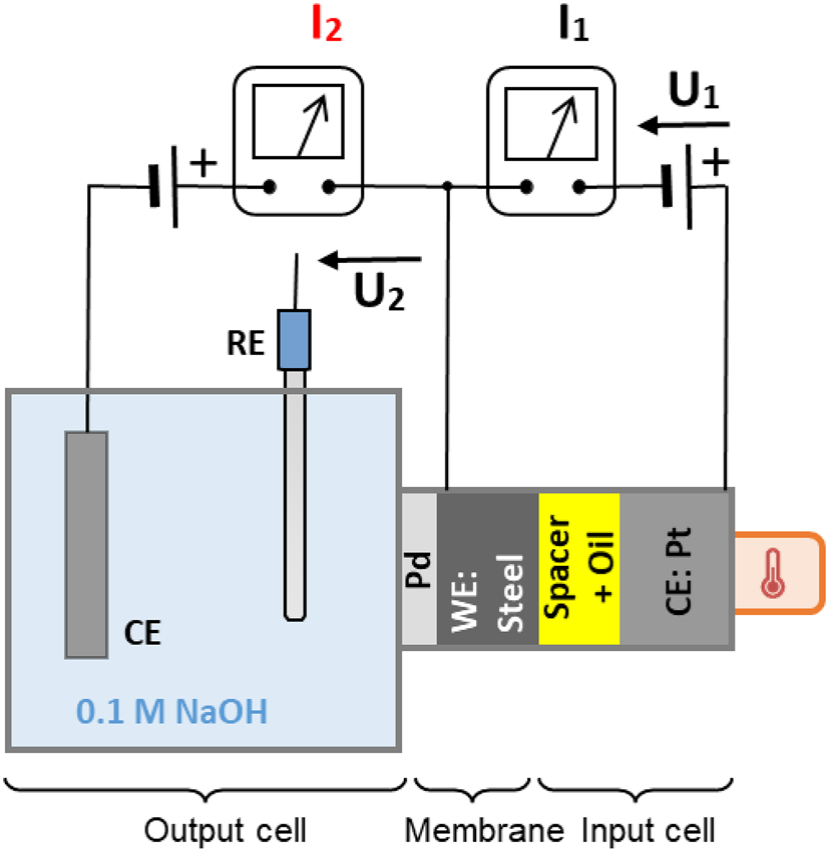
Schematic representation of the modified Devanathan–Stachurski cell. *RE* reference electrode, *CE* counter electrode, *WE* working electrode.

Schematic representation of the modified Devanathan–Stachurski cell with a close steel/Pt connection in the input cell is given in Fig. 1.

The "model compositions” of Base oil and additives tested by the modified Devanathan–Stachurski cell in order to demonstrate its application for investigation of the hydrogen permeation process into a steel membrane under cathodic polarization are presented in Table 1.

**Table 1:** Model oil compositions and water content determined by Karl Fischer titration.

Oil composition	Abbreviation	Water content/ppm
Ester base oil (Trioctyl trimellitate)	BOil	318
BOil + 1% Bis(nonylphenyl)amine (Antioxidant, AO)	BOil + AO	361
BOil + 15%Zinc-dialkyldithiophosphate (ZDDP)	BOil + ZDDP	2800
BOil + 15% Calcium Sulfonate (CaSulf)	BOil + CaSulf	1800
BOil + 1%AO + 2%ZDDP + 15%CaSulf	CombOil	778
BOil + 1%AO + 2%ZDDP + 15%CaSulf + H_2_O	CombOil + H_2_O	6516

The chemistry of the model oil compositions is common for lubricants. Bis(nonylphenyl)amine is a typical antioxidant. ZDDP is widely referred as extreme-pressure/anti-wear additive and CaSulf as detergent/rust preventative. The concentrations of ZDDP and CaSulf are significantly higher than that in industrial oils aiming to clearly distinguish possible effects of the additives on the hydrogen generation and permeation.

In order to achieve high sensitivity in the detection of diffusible H generated by polarization of the oils, mild steel with a thickness of 25 µm (Metall-Folien GmbH, Main, Germany) was chosen instead of bearing 100Cr6 steel. The thickness of the steel electrodes is a critical parameter. 100Cr6 steel in the desired quality in bearings, i.e. heat treated, 62 ± 1 HRC with a specified roughness, surface flatness, thickness and dimensions for the hydrogen permeation setup is not commercially available. It could be assumed, that the different hydrogen trapping sites and the different hydrogen diffusivity of the two steels should not greatly affect the conclusions on the effect of cathodic polarization of lubricating oils on their ability to generate hydrogen as well as for the method validation.

Before experiments, the steel electrodes, prepared from a mild steel foil with a thickness of 25 µm were cleaned by degreasing in NaOH solution and acetone, followed by etching in aqueous solution of HCl (1:1). The working surface area was 1 cm^2^.

The spacer of filter paper was preliminary dried (at 150 °C for 30 min in a laboratory oven) and placed on the platinum counter electrode**.** Thereafter, the filter paper was impregnated with the oil tested (0.5 ml). After assembling the input cell, the exit side of the steel membrane was electroplated with a thin (nearly 2 µm) palladium layer.

0.1 M NaOH solution was introduced into the output cell. Constant positive potential of 0.28 V vs. Hg/HgO was applied on the exit side of the steel membrane. The detected oxidation (permeation) current I_2_ is proportional to the amount of diffusing hydrogen through the steel membrane. By a computerized system, the current I_2_ at the exit side of the steel membrane was recorded against time as a measure of the hydrogen penetrated into the steel membrane (hydrogen permeation transient).

Before recording the permeation transient, a positive potential of 0.28 V vs. Hg/HgO was applied on the exit side of the steel membrane for a sufficiently long time to oxidize hydrogen resulting from its prior treatment (manufacturing process, Pd plating) and to get a residual anodic current density (I_2_^res^) less than 2 µA/cm^2^.

In the case of experiments at room temperature, when reaching I_2_^res^ less than 2 µA/cm^2^, a voltage of 30 V (Fig. 1, U_1_) was applied on the entry side of the membrane by a programmable power supply Instek, PSP-405. The 30 V polarization is in agreement with reports in the literature (the bearing voltage can reach ~ 30 V^34^ and be higher than 70 V^22^). The reference value of the current at 30 V polarization at room temperature is in the 15–20 nA range, while at heating the current value is within the 0.8–1.7 µA range, depending on the oil composition and temperature. Upon heating, at first the heating device was switched on, set to 70 or 100 °C. After at least 40 min (which time was sufficient the system to be heated), the voltage of 30 V was applied. At the end of the experiment, first the voltage was switched off and then the heating device. The temperature was measured close to the platinum counter electrode, using a thermo-couple mounted within the input cell. Generally, in application the bearing temperature in lubricated contact can be 60–90 °C, though localized flash temperatures can be much higher^31^. In the present work the effect of temperature of 70 °C (within the operational range) and of 100 °C (upper temperature limit due to evaporation of the electrolyte in contact with the steel at the exit side of the membrane) on the hydrogen permeation was investigated. The increase of the temperature affects the viscosity and the electrical conductivity of the oil, depending on its composition and greatly affects the hydrogen diffusion through steel.

Karl Fischer titration was used for determination of the water content in the BOil and in the oil compositions at room temperature. Both additives, ZDDP and CaSulf are known to have hygroscopic properties. Artificially, water was added to the BOil and the CombOil to investigate its effect on hydrogen permeation and electrical conductivity of the substances.

Electrochemical impedance spectroscopy (EIS) was employed to measure the electrical resistance (impedance) of the steel membrane/oil interface within the 5–2 MHz range of frequencies. The results obtained in EIS for the low-frequency region were used to evaluate the effect of the water content on the oils resistance. The EIS measurements were performed in 2-electrode configuration at room temperature. Electrodes of mirror polished stainless steel with a distance in-between of 50 µm (defined by PTFE foil) and working area of 1 cm^2^ (exposed to the oil) were used.

### Ethical approval

Each author certifies that his or her institution approved the study protocol and all investigations were conducted in conformity with ethical principles of research.

## Results and discussion

### Hydrogen permeation measurements with base oil (BOil). Influence of the temperature

**Figure 2:**
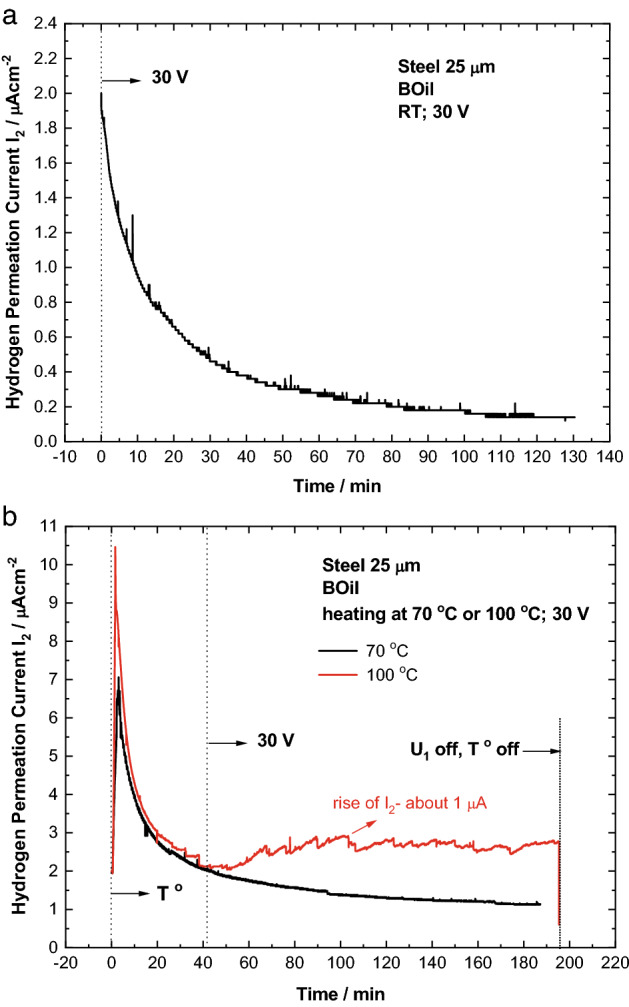
Hydrogen permeation transients, recorded with BOil under voltage of 30 V at room temperature (**a**); upon heating at 70 °C or 100 °C (**b**).

The transient of BOil, obtained at room temperature and under constant voltage of 30 V is shown in Fig. 2a. As soon as the residual current I_2_^res^ reached the value of about 2 μA, the steel membrane was subjected to polarization. No rise of the permeation current is observed. On the contrary, the permeation current continues to decrease. Upon heating at 70 °C or 100 °C (Fig. 2b), the corresponding transients of BOil begin with a sharp increase of the permeation current I_2_. After this peak, the voltage of 30 V was applied. There is no influence of the polarization on the permeation current at 70 °C. However, upon heating at 100 °C, the transient shows an increase of I_2_ of about 0.8 μA. This effect can be related to the simultaneous impact of both factors: heating (influencing the oil conductivity, and the dissociation, surface and diffusion kinetics) and polarization (formation of atomic hydrogen) on the hydrogen permeation behavior.

It should be noted that all the permeation transients obtained upon heating begin with such a sharp increase of the hydrogen permeation current I_2_ further named “temperature peak”, because of its attribution only to the increased temperature. The initial “temperature peak” depends on the temperature and it is higher at 100 °C compared to 70 °C.

Concerning the nature of the “temperature peak”, it can be associated with release of residual trapped hydrogen already present in the steel membrane from its manufacturing process and hydrogen resulting from the preliminary treatment of the steel membrane (etching and Pd plating). Such a peak was not registered when experiments with Pd working electrode were carried out, suggesting that the maximum is due to hydrogen, which is not evolved from the steel membrane at room temperature prior heating of the system. A part of the hydrogen (diffusible hydrogen) diffuses and oxidizes due to the applied positive potential at the exit side of the membrane at room temperature, reaching I_2_^res^ value of about 1–2 μA. The subsequent increase of the temperature, on one hand changes the concentration of diffusible hydrogen by the exchange reaction with the traps in the steel; on the other hand, the value of the diffusion coefficient increases. Both effects contribute separately to the measured increase of the hydrogen oxidation current I_2_.

It is well known that metallic materials have defects in their atomic lattice structure, e.g. due to the manufacturing process. After absorption, hydrogen diffuses into the microstructure of the metal lattice and there, it is distributed in two forms: diffusible and trapped hydrogen. The trapped hydrogen is incorporated in the micro-structural in-homogeneities (interfaces, voids, grain boundaries, dislocations, micro-cracks and blisters)^35^. Depending on the binding energy of hydrogen to the trapping sites, trapped hydrogen can be classified as reversible or irreversible^36–38^. Hydrogen trapping and hydrogen diffusion, both processes are temperature dependent.

### Hydrogen permeation measurements with combined oil (CombOil). Influence of the temperature

**Figure 3:**
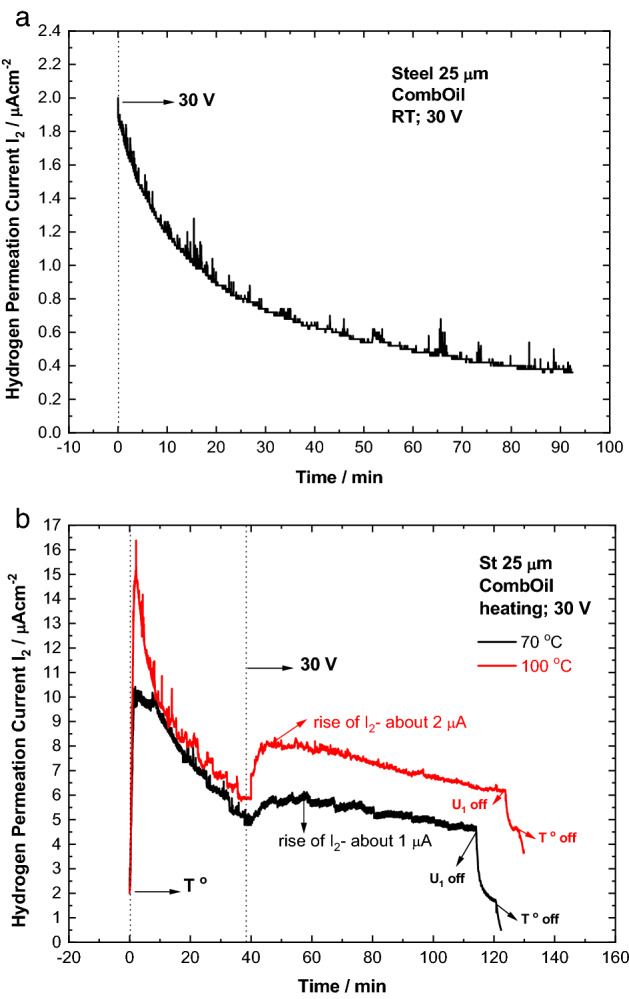
Hydrogen permeation transients, recorded with CombOil under voltage of 30 V at room temperature (**a**) and upon heating at 70 °C or 100 °C (**b**).

Similar to BOil, in the case of CombOil, a decreasing trend of of I_2_ is observed on the hydrogen permeation transient recorded under voltage at room temperature (Fig. 3a). Compared to BOil the current transient of CombOil shows slightly more noise.

On the transients of CombOil obtained upon heating at 70 °C or 100 °C (Fig. 3b), an increase of I_2_ is observed on both transients after applying the voltage. Compared with BOil, the transient of CombOil shows an increase of I_2_ under voltage even at the lower temperature of 70 °C. Comparison between the transients of CombOil and BOil upon heating at 100 °C shows higher increase of I_2_ under voltage (about 2 μA) at the transient of CombOil, i.e. more than twice than that registered on the transient of BOil. The observed additional increase in the permeation current on the transient of CombOil under polarization can be related to the presence of additives (and/or water contamination) in the CombOil. Therefore, in the case of CombOil, simultaneous action of the three factors (polarization, heating and additives) on hydrogen permeation behavior of the oil is registered.

### Influence of individual additives to the base oil on the hydrogen permeation current upon heating

**Figure 4:**
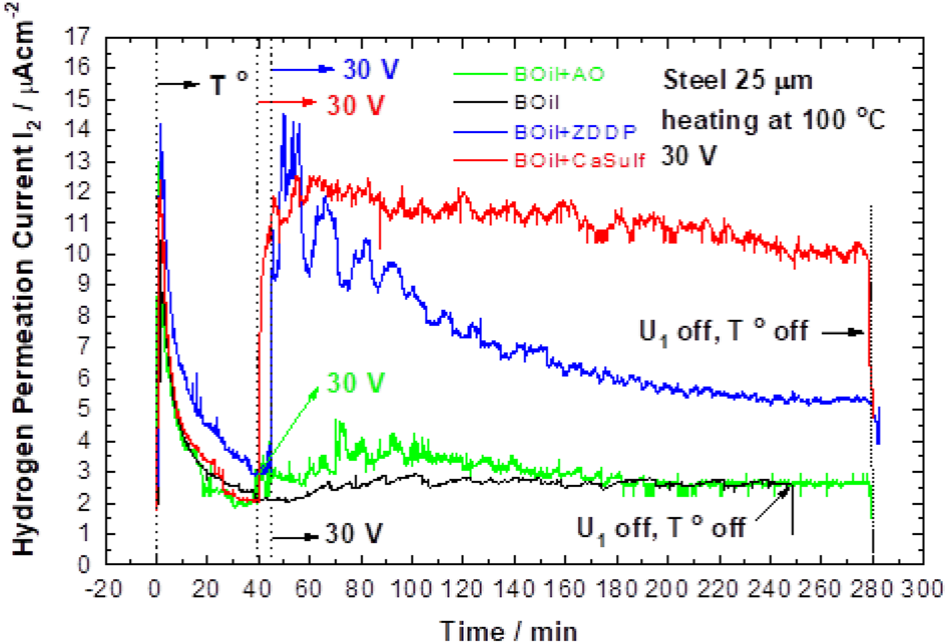
Comparison of transients, recorded under voltage of 30 V and upon heating at 100 °C with BOil and the compositions BOil + AO; BOil + ZDDP or BOil + CaSulf.

The transient corresponding to BOil shows the lowest activity of the oil in regard to the hydrogen permeation under polarization (Fig. 4). However, all the combinations of BOil with the individual additives show some increase of the hydrogen permeation current I_2_ under the applied voltage.

The addition of the antioxidant (AO) to the BOil slightly increases the hydrogen permeation activity of the BOil. The presence of the additives ZDDP or CaSulf into the oil compositions (BOil + ZDDP or BOil + CaSulf) leads to very strong increase of the hydrogen permeation current I_2_ immediately after applying polarization, the effect being more pronounced in the case of BOil + CaSulf. The trends of hydrogen permeation transients for BOil + CaSulf and BOil + ZDDP are also different. For BOil + CaSulf the permeation current slightly decreases with time, while that of BOil + ZDDP remarkably decreases with time. These suggest differences in the mechanism of action of the additives regarding the hydrogen permeation in steel.

Considering the water contamination as an only source of hydrogen under polarization does not explain the higher permeation current registered for BOil + CaSulf, the composition with a lower water content than that of BOil + ZDDP (Table 1). Therefore, an effect of the chemical composition of the additives is to consider, as well.

It could be proposed that the presence of sulfur in the CaSulf additive greatly influences the rate of hydrogen permeation into the steel for BOil + CaSulf. As rust preventative additive, it could be assumed that CaSulf is present at the steel surface. Cathodic polarization might lead to partial reduction of the sulfonate to R–S^−^ species^30^. Sulfur at the steel surface is a poison for the recombination of atomic hydrogen to molecular, which would increase the surface concentration of atomic hydrogen. The hydrogen permeation current is proportional to the absorbed hydrogen and in correlation with the hydrogen concentration gradient (the hydrogen concentration at the surface at the entry side of the membrane and the concentration at the exit side of the membrane, which is zero due to the applied positive potential). It is widely discussed in the literature that the hydrogen poisoning sulfur can promote hydrogen permeation by inhibiting molecular hydrogen recombination in aqueous systems^1,3,6–14^ and non-aqueous^20,30,31^ systems.

The ZDDP additive contains both critical for hydrogen absorption elements, sulfur and phosphorous. This suggests similarity in the course of the permeation transient for BOil + ZDDP to that of the BOil + CaSulf, if the same mechanism is assumed. However, the relatively high permeation current reached shortly after the polarization of BOil + ZDDP is followed by a decay. Formation of a layer at the steel surface inhibiting the hydrogen absorption could be a possible reason for this. Research on ZDDP highlights that ionic species formed during redox reactions will tend to be insoluble in non-aqueous liquids and, unless removed physically, will tend to block the electrochemical processes^30^. At the condition of cathodic polarization, discharge of Zn ions and local deposition of Zn on the steel surface could also take place. Zn metal is unable to adsorb hydrogen atoms to any great extent^40^ and would serve as an effective barrier to hydrogen absorption, resulting in a decrease of the permeation current. Preliminary SEM and EDX analysis of the steel surface performed after hydrogen permeation tests in this research showed formation of a discontinuous layer and presence of Zn at the steel surface [in the range of 1–6 mass % (integral analyses)] among Fe, O, C, S and P. The images and the data from the surface analyses obtained after the permeation tests and their comparison with the penetration currents will be a subject of a separate manuscript.

By in situ H-detection setup, developed on the basis of dynamic modification of Devanathan–Stachurski cell, the anti-wear additive ZDDP is confirmed to play a role of promoter on hydrogen uptake into steel during lubricated tribo-contact^27,28^. It is supposed that ZDDP exerts inhibition effect on the recombination of hydrogen atoms to hydrogen gas. As a result, the concentration of absorbed atomic hydrogen in the metal is increased. According to Refs.^28,39,41^, the products formed by hydrolytic decomposition of ZDDP (Alkyl sulphides and Zinc-polyphosphates) have a role of protection of the engine, but they promote the hydrogen permeation process.

**Figure 5:**
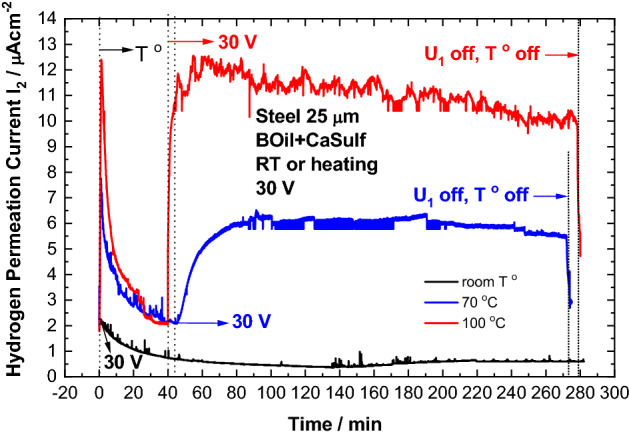
Hydrogen permeation transients recorded with BOil + CaSulf under voltage of 30 V at room temperature or upon heating at 70 °C or 100 °C.

The influence of the temperature is demonstrated by the hydrogen permeation measurements with the composition BOil + CaSulf under polarization at room temperature as well as upon heating at 70 °C or 100 °C (Fig. 5).

Very low activity of the oil is registered at room temperature. Strong increase of the hydrogen permeation current under voltage is registered upon heating, the effect being more pronounced at 100 °C. This could be related to a release of water from hydrates of the hygroscopic CaSulf at elevated temperatures. It is well known, that at temperatures above 60 °C weakening of the hydrogen bonds in hydrates takes place.

Both, Ca-sulfonate and Zinc dialkyl-dithiophosphate have a hygroscopic effect. Data for the promoting effect of water contamination in lubricants are published in Refs.^27,28^. It is proven in Ref.^28^, that hydrogen entry into the steel plate is strongly promoted by the additives or water in the lubricant PAO at a lubricated rubbing contact. In addition, a synergistic effect between ZDDP and water is observed—the promoting effect of ZDDP + H_2_O is twice more than that of ZDDP or H_2_O separately. Two hypotheses are stated for the promoting effect of water: the surface protective layer formed from ZDDP is removed by water or water promotes surface corrosion and accelerates hydrogen formation as a corrosion product.

The effect of artificially added water to the lubricating oil is demonstrated in the next section.

### Influence of water content in the CombOil on the hydrogen permeation current

Water contamination of lubricating oils is considered as one of the prime sources of hydrogen in a lubricating tribo-system. The water enters the lubricating oil during operation of the bearing either from humid air in contact with the lubricant or by condensation of the water in the system. The water absorbing capacity of the lubricant varies depending on its chemical composition.

Deleterious effects of water contamination on the bearing fatigue life were well studied^23,27,28,42–45^. According to the literature data, even low water concentrations are sufficient to dramatically reduce the service life of steel. It was established that the lubricant transports the water directly to the steel surface where it dissociates due to the heat released by the friction^46^. The dissociated water is reduced to hydrogen at the nascent steel surface and additionally promotes the oxidative decomposition of lubricants^25^. The hydrogen diffuses into the steel matrix, causing the initiation of hydrogen embrittlement and WEC.

In the present work, the influence of water (hydration water, associated with the oil composition or artificially added) for the CombOil on the hydrogen permeation into steel membrane subjected to cathodic polarization was investigated.

**Figure 6:**
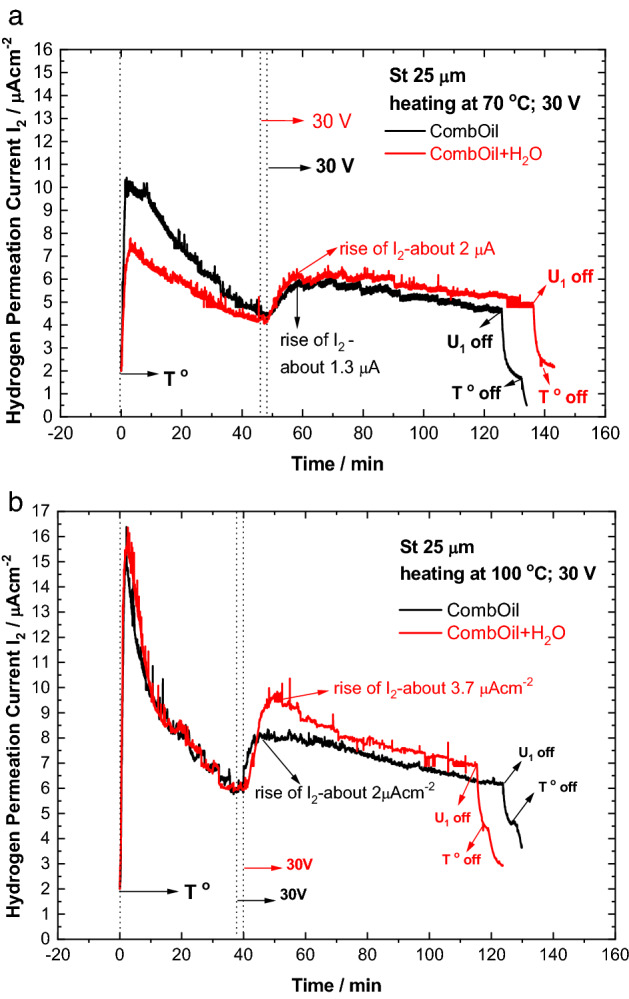
Hydrogen permeation transients recorded with CombOil and CombOil + H_2_O under voltage of 30 V and upon heating at 70 °C (**a**) or 100 °C (**b**).

Figure 6 shows the compared transients of CombOil and CombOil + H_2_O recorded upon heating at 70 °C (Fig. 6a) and at 100 °C (Fig. 6b).

At both temperatures, the increased water content in the oil substance leads to an increase of the hydrogen permeation current. As seen, the increase of the hydrogen permeation current at 100 °C is higher than that measured at 70 °C.

**Figure 7:**
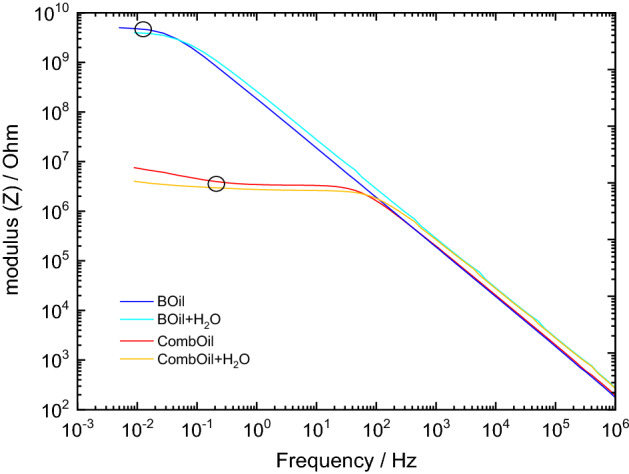
Electrochemical impedance spectra for BOil, BOil + H_2_O, CombOil and CombOil + H_2_O at room temperature.

The effect of water addition on the hydrogen permeation transients is not as significant as expected. On the one hand, the water is a source of hydrogen and on the other hand, affects the electrical conductivity of the substance. Therefore, electrochemical impedance spectroscopy was employed for investigating BOil, BOil + H_2_O and CombOil, CombOil + H_2_O at room temperature (Fig. 7). As seen in Fig. 7 in the range of the low frequencies the modulus of the impedance for the CombOil and CombOil + H_2_O are similar, while these values are about three orders of magnitude lower than that for BOil and BOil + H_2_O. This indicates much stronger effect of the additives (and the associated hydration water) on the electrical conductivity, then that of water content itself.

## Summary

A modification of the Devanathan–Stachurski cell is developed, aimed to achieve very close distance between the working electrode (steel membrane) and Pt counter electrode in the input cell using a spacer of filter paper. By this way, the ohmic resistance of the input cell is lowered sufficiently to allow in situ hydrogen permeation experiments with lubricating oils. Investigations are performed under constant voltage of 30 V at room temperature or upon heating with various oil substances.

The following regularities are found:

- Subjecting the lubricating oils to cathodic polarization results in generation of hydrogen diffusing through the steel membrane. At a higher temperature, a stronger effect of polarization of the oils on the permeation current is observed.
- Under polarization and upon heating, the hydrogen entry into the steel membrane is promoted by the additives tested (especially ZDDP or CaSulfonat) as well as by the presence of water in the lubricating oils. The effect of the additives on the hydrogen permeation and on the electrical conductivity of the oils is much stronger than that of water content itself.

The phenomenon hydrogen permeation into steel membrane from lubricating oils subjected to cathodic polarization is obviously related to the complex impact of several factors—temperature, additives and presence of water in the oil substances.

The modified Devanathan–Stachurski setup can be used as a method for in situ evaluation of the lubricating oils (and ingredients) in respect to their ability to generate hydrogen under cathodic polarization. This finding is fundamental for further investigations on the effect of external polarization of lubricants on hydrogen generation and permeation in steel in a lubricated tribo-contact. By further modification of the set up the tribological load of the lubricants will be also examined.

## Data Availability

The data that support the findings of this study are available from the corresponding author upon reasonable request and with permission of Klüber Lubrication München GmbH & Co. KG.
